# The Management of Ureteropelvic Junction Obstruction Presenting with Prenatal Hydronephrosis

**DOI:** 10.1100/tsw.2009.51

**Published:** 2009-05-29

**Authors:** C.D. Anthony Herndon, David M. Kitchens

**Affiliations:** University of Alabama at Birmingham, Birmingham, USA

**Keywords:** pediatrics, ureteropelvic obstruction, hydronephrosis

## Abstract

The treatment of the newborn diagnosed with a ureteropelvic obstruction prenatally should follow a systematic approach. Although a majority of patients can be followed without surgical intervention, controversy exists concerning appropriate follow-up. Furthermore, a significant number of patients will manifest mild disease and thus deserve abbreviated follow-up. Herein, an appropriate algorithm and a review of the literature are discussed.

## INTRODUCTION

Ureteropelvic junction (UPJ) obstruction represents the most common diagnosis seen in infants detected prenatally with hydronephrosis. The majority of infants detected prenatally will be followed without surgical intervention. Thus, UPJ obstruction is a spectrum of disease. The postnatal evaluation follows a set protocol that involves both ultrasound and nuclear medicine imaging. Surgical intervention will be required for the most severe form of UPJ obstruction in 20% of patients. Fortunately, it is successful in most patients after one surgical procedure. Recently, the use of minimally invasive techniques, such as laparoscopy with or without robotic assistance, has become more common. Postoperative complications are uncommon and long-term prognosis is excellent.

## NEWBORN IMAGING AND SURGICAL REPAIR

Imaging of the newborn kidney will be influenced by hydration status; therefore, the initial renal ultrasound is recommended on day 2 of life. Repeat imaging is based on the initial ultrasound, but a majority of patients will be re-evaluated at 1 month with a repeat ultrasound and/or voiding cystourethrogram (VCUG). The utilization of renal scintigraphy is individualized, but will provide useful information in terms of relative renal function as well as upper urinary tract drainage. Recommendations for surgical intervention are rarely made based on one set of images. Rather, a decision to proceed towards surgical intervention is based on serial imaging and documentation of a failure to improve, worsening of hydronephrosis, or deterioration in renal function. Patients that become symptomatic with colic, urinary tract infections, stones, and hematuria may also be candidates for surgical intervention.

The initial enthusiasm for early intervention for UPJ obstruction that was recommended in the early 1980s has evolved to a more conservative approach[1,2,3,4]. The most compelling data to support an initial interval of observation are those found in the long-term follow-up by Koff and colleagues. In this study, 104 newborns with unilateral severe hydronephrosis were managed expectantly. A deterioration of renal function was the indication for surgical repair. A total of 23 patients required intervention and a return of function was appreciated in all of these patients. In addition, hydronephrosis resolved in 69% and improved in 31%. The average time to this resolution was 2.5 years[4].

The initial report from the Society for Fetal Urology (SFU) focused on 33 surgeons representing 21 centers that included 582 cases of antenatal hydronephrosis. Intervention was required in 41% of patients. In the intervention cohort, most patients demonstrated an obstructed renal scan and SFU grade 3 or 4 hydronephrosis. For the nonintervention cohort, grade 1 and 2 hydronephrosis predominated[5]. Although a consensus appeared to exist on characterizing those patients that underwent intervention, controversy still remained in identifying which of the patients with SFU grade 3 and 4 hydronephrosis would go on to need intervention.

The most comprehensive series to date is the Great Ormond Street experience of prenatal hydroneprhosis (PH). In a two-part series, they initially followed a standard protocol from 1980 to 1988[6]. Patients with PH were divided into three groups based on differential renal function alone: <20%, 21–39%, and ≥40%. In the first group (<20% differential renal function), three out of 10 kidneys that initially underwent pigtail drainage were successfully managed with surgical correction. In the second group (21–39% differential renal function), 23 out of 28 underwent immediate pyeloplasty with 12 demonstrating an improvement in differential function and a stabilization of differential function in the remaining patients. The five patients that underwent observation, all improved on long-term follow-up without surgical intervention. For the last group (≥40%), none of the 34 kidneys with less than 12-mm pelvic dilation on the postnatal ultrasound required surgical intervention, compared to 27 of the 76 kidneys with pelvic dilation greater than 12 mm that went on to have surgical correction of the obstruction.

Based on their observations, they set up a randomized trial, conservative vs. operative. The inclusion criteria were an anteroposterior renal pelvic diameter > 15 mm postnatally, calyceal dilation, and differential function of ≥40%. Seventy-five patients (53 boys) were enrolled. Both groups were comparable in terms of mean hydronephrosis and differential function. At last follow-up, 39 patients underwent successful surgical intervention demonstrated by a decrease in hydronephrosis and an improvement in drainage. The mean function post-op of 50% compared favorably to the pre-op mean of 48%. One patient demonstrated a decrease in function from 43 to 38%. For the conservative group, 36 patients were randomized. Hydronephrosis resolved in 17 and improved significantly in 12. Surgery was required in seven infants for renal deterioration, which was recoverable in all but one patient. Again, anteroposterior diameter appeared to predict those patients requiring intervention. All seven of the patients that crossed over to the intervention group demonstrated an anteroposterior diameter of >20 mm (20–40). However, nine out of 17 of the patients with spontaneous resolution also initially demonstrated an anteroposterior diameter of >20 mm.

Relying solely on radiologic imaging may not allow adequate prediction of those renal units amenable to reconstruction. Elder et al. compared renal biopsy data to preoperative renal scan differential function data[7]. Interestingly, in the group with less than 40% differential function, 25% demonstrated essentially normal findings on renal biopsy. In the group with normal renal function, 21% demonstrated abnormal renal histology. Based on these data, caution must be utilized when interpreting differential function based on the well-tempered renal scan.

Traditionally, the surgical repair of UPJ obstruction has involved an open surgical procedure that was performed retroperitoneally. The two surgical approaches involve either a flank or dorsal incision, and success rates have approached 100%[8,9,10]. Recently, a mini-incision approach has been described with equal results when compared to a more traditional standard incision[11]. However, laparoscopy with and without robotic assistance has become more prevalent for neonatal and infant UPJ repair[12]. Vemulakonda et al. reviewed the pediatric health information system database and noted an increased percent, from 2.5 to 9.7%, of pyeloplasties performed laparoscopically over a 2-year interval. After this time period, the percent of laparoscopic pyeloplasties plateaued[13]. One issue that has been raised is what impact a transperitoneal procedure that involves opening the urinary tract would have on surgical outcome. Piaggio et al. reported a retrospective review that compared outcomes of an age-matched control in a laparoscopic and open pyeloplasty cohort. They demonstrated a decrease in complication rate in infants older than 14 months in the laparoscopic group ($p < 0.05$) and a decrease in surgical time after 15 cases ($p < 0.001$)[14]. Neheman et al. demonstrated no difference in outcomes or complication rate in infants weighing less than 10 kg when treated with transperitoneal laparoscopic pyeloplasty in comparison to a control open cohort[15,16]. Although concerns for encroachment on the peritoneum are valid, the results from the transperitoneal procedure make them unfounded. Length of stay and narcotic requirement were no different. Canon et al. compared a retroperitoneal vs. transperitoneal laparoscopic cohort and found no difference in outcome, but a significantly longer mean operative time in the retroperitoneal cohort. Based on these data, they have endorsed a transperitoneal approach[17]. Olsen et al. reviewed their 5-year series of 67 retroperitoneal robotic pyeloplasties and found an initial surgical success of 94%. They felt that retroperitoneal access facilitated the repair because of its direct access to the UPJ[18].

## CONCLUSION

A practical approach should be followed when caring for the neonate with PH. All patients that are considered candidates for intervention should be placed on amoxicillin prophylaxis, 2 cc (125 mg/cc) nightly. In cases of grade 4 hydronephrosis, a repeat ultrasound and “well-tempered” renogram should be performed at 1 month. In the select case of profound delay in drainage or a demonstrative decrease in differential function, immediate intervention should be employed. In most patients, intervention can be deferred and a repeat ultrasound obtained at 3 months of age. If the renal ultrasound indicates worsening or shows increased signs of distention, then a repeat “well-tempered” renal scan should be performed. Surgical intervention is indicated for an obstructive drainage curve or indeterminate curve with a deterioration in renal function (<40%). Close follow-up is mandatory for grades 3 and 4 hydronephrosis that are managed without surgical intervention.

Surgical intervention will be required in 20% of patients, with a majority of those representing grade 3 or 4 hydronephrosis. Traditionally, open surgical repair has been the gold standard, but recently laparoscopy has become increasingly prevalent. Laparoscopy is still in its infancy in terms of access and utilization of robotics. When comparing all modalities of retroperitoneal and peritoneal approaches, as well as with and without robotics, outcomes appear to be similar to the open procedure. A clear trend is presently in favor of a minimally invasive approach that now is being utilized in the neonatal population. Complications do occur, but are not common with either modality and long-term prognosis is excellent.
